# Settings for the development of health literacy: A conceptual review

**DOI:** 10.3389/fpubh.2023.1105640

**Published:** 2023-02-16

**Authors:** Catherine L. Jenkins, Jane Wills, Susie Sykes

**Affiliations:** Institute of Health and Social Care, London South Bank University, London, United Kingdom

**Keywords:** health promoting settings, supersetting approach, systems, health literacy, settings approach

## Abstract

Advances in conceptualizing settings in health promotion include understanding settings as complex and interlinked systems with a core commitment to health and related outcomes such as health literacy. Traditional settings for the development of health literacy include health care environments and schools. There is a need to identify and conceptualize non-traditional and emerging settings of twenty-first-century everyday life. The aim of this conceptual review is to inform a conceptual model of a “non-traditional” setting for the development of health literacy. The model uses the example of the public library to propose four equity-focused antecedents required in a setting for the development of health literacy: the setting acknowledges the wider determinants of health, is open access, involves local communities in how it is run, and facilitates informed action for health. The review concludes that a settings approach to the development of health literacy can be conceptualized as part of a coordinated “supersetting approach,” where multiple settings work in synergy with each other.

## 1. Introduction

The World Health Organization has long recognized that settings can be health-promoting or health-threatening: “[i]f health is everywhere, every place or setting in society can support or endanger health” (1) (p. 65). The Ottawa Charter for Health Promotion (2) views health as “created and lived by people within the settings of their everyday life; where they learn, work, play and love” (2) throughout the life course (3). “Settings for health” is used in this review as defined in the latest edition of the Health Promotion Glossary of Terms (4) (p. 30):

The place or social context where people engage in daily activities, in which environmental, organizational and personal factors interact to affect health and wellbeing.

Health literacy is a domain of health promotion and enables people to access, understand, appraise, remember, and use information about health (5). Health literacy can be developed through interaction with, and is influenced by, settings such as health care environments (5) and schools (6) but there is increasing recognition that it can also be developed in “new” and emerging settings for health, such as online settings (social media and virtual reality) and hybrid settings (settings with physical and online manifestations; augmented reality) (7–11).

One of the Ottawa Charter's action areas focuses on creating “supportive environments” for health, but the Charter has not formally kept pace with the expansion of everyday settings for health (12) to include e.g., online settings (13), or “where people google” (14). More recently, the World Health Organization has referred to “enabling environments,” which “support people to access, understand, appraise, remember and use information about health” (i.e., develop health literacy), “for the health and wellbeing of themselves and those around them, within the circumstances and demands of their daily lives” (15). A rapid review of settings for raising awareness of health inequities (16) has suggested several types of non-traditional settings for health: online, faith-based, night-time economy, green (“eco”) and temporary pop-ups. Reviewing the settings approach from alternative angles and categorization, e.g., by opening hours, ecological footprint, and permanence, can revitalize the evidence base and ensure that it retains relevance.

The supersetting approach, or “settings approach 2.0” (17), is one such revitalization. It is a multisetting approach to health that emphasizes “the need for coordinated activities to be carried out in a range of different settings within a local community with the aim of attaining synergistic and sustainable effects” (3) (p. 30–31). This paper outlines a conceptual review of settings in the context of the settings approach in health promotion. The conceptual review informs a conceptual model of an example “new” setting, the public library, as a community-based setting for health and health literacy when part of a wider supersetting approach. The public library is selected as an illustrative example with four antecedents conducive to health literacy development. The antecedents are reported in more detail elsewhere (18); this review focuses specifically on the relevance of the settings concept to health literacy.

Community-based and informal education settings broadly accessible to people include “extended classrooms” such as “parks, shopping centers, community centers, or libraries” (19) (p. 146). The public library is the example case used in this paper, for several reasons. Within the field of public health, public libraries constitute a comparatively unique but under-utilized community partner, particularly in rural areas (20–22). Public libraries' reach is inclusive of otherwise marginalized communities, such as school-excluded children and the homeless. Conceptualizations of public libraries as responsive and active community hubs for health-related activities and information-seeking highlight their provision of flexible physical and virtual space, informal learning opportunities, and curation of free access to local and global health information services (23, 24). Library-based resources include the staff, some of whom will be trained information professionals with an understanding of health communication, infodemiology, and the determinants of health relevant to the communities they serve (25). Many public libraries have a consumer health librarianship function (26) and “routinely assist patrons with unmet health and social needs” (27) (p. 1).

Despite evidence internationally in support of the potential role of the public library in public health and, to some extent, health literacy (23, 28, 29), this setting constitutes a missed opportunity: it is not considered a “traditional” setting for health (30). The current conceptual review therefore uses the public library as an example to explore the potential of non-traditional or emerging settings for health and health literacy.

## 2. Methods

The importance of settings as a concept for the promotion of health is longstanding and there is a significant body of literature that explores the concept (31) and its practice (3), and yet its theoretical basis is contested (32, 33). A conceptual review examines the discursive scaffolding of a concept and contributes more nuanced understandings of the connections between that concept and empirical evidence (34). Revisiting the literature *via* a conceptual review can foster “revitalization of existing theory,” or even “novel conceptual insights” (35) (p. 28).

A conceptual review of the settings-based approach was conducted using a systematic process of searching across databases and gray literature, and reading the retrieved literature critically to map and clarify this concept in its historical and social context (34). While the review was conducted systematically, it differs significantly from a systematic review. One such difference is the way in which a conceptual review is reported: there is no extension to PRISMA available for the conceptual review type, and therefore conceptual review reporting tends to be discursive in nature. The process incorporated five stages: establishing the parameters of the concept under review, integrating and synthesizing the evidence base (both conceptual and empirical), resolving inconsistencies and tensions, highlighting gaps in the existing literature, and outlining an agenda for future research (35).

The first stage involved defining the settings-based approach in health promotion and distinguishing it from related concepts by formulating and applying eligibility criteria to separate out instances of conceptual conflation and terminological confusion. The second stage used citation analysis of canonical or pertinent sources (3, 13, 17, 31, 33, 36) to comprehensively trace the development of the settings concept and its theorization. Inconsistencies and ambiguities, e.g., between definitions and operationalizations of the concept, were resolved systematically by grouping the amassed evidence into research “streams” that could be examined side-by-side (35). This examination led to the next stage: gap or “tensions” analysis, focused specifically on where an absence of evidence or the presence of tensions limited the ability of the settings-based approach to evolve and respond to twenty-first-century determinants of health. The conceptual tensions identified are reported in the Results.

## 3. Results

The results of the conceptual review are presented as themes that each reveal a tension in the narratives around settings and ways in which the concept has been clarified or developed, such as using complexity theory to represent settings as systems. Overlapping concepts identified from the literature are used to organize the results based on how the public library setting is understood: as a “setting for health,” a “system,” a “health-literate organization,” or part of a supersetting approach. The results inform a conceptual model of the public library as part of a supersetting approach.

### 3.1. Settings for health and health literacy

The conceptual review provides insight into what is known about “settings.” In health promotion policy and literature, “setting” is used in two ways: health promotion *in* a setting (where the setting serves as the location for individually-oriented lifestyle interventions), and settings-based health promotion (where the setting *is* the health promotion intervention) (3, 32). In the 2021 update to the Health Promotion Glossary of Terms (4), under a new entry for “environmental determinants of health,” settings for health are referred to as providing the “structure for practical action” (p. 15). Action also appears under the “settings for health” entry itself, where the indicator that “people actively use and shape the environment” differentiates settings for health from “a setting as the basis for delivery of a specific service or programme” (p. 30).

The emphasis on “traditional” settings for health—“healthy cities; health promoting schools; healthy workplaces; healthy islands; health promoting hospitals; health promoting prisons and health promoting universities” (4) (p. 30)—can circumscribe applications of the settings-based approach. This selectivity is beginning to change with the induction of non-traditional settings, such as healthy stadia and airports, into the evidence base, “some through formalized initiatives led by the WHO and other bodies, others emerging through pilot studies and projects” (33) (p. 12); the public library is an ongoing example of the latter route (18, 21, 29).

### 3.2. Settings as systems

The settings-based approach understands settings as complex systems with inputs, throughputs, outputs, and impacts (32) in relation to a wider environment (including other settings). This complexity requires drawing on “multiple theories from multiple disciplines, rather than one overarching theory” (32) (p. 15) to consolidate knowledge of how settings-as-systems work. In the UK and other countries, the public library is organized and referred to as a “system” of distributed local branches.

Socioecological models identified in the conceptual review span theoretical stances and include a model of a non-traditional setting (a sports club) that shows the reciprocal interaction between setting- and individual-based factors for health at macro-, meso-, and micro-levels (37), and a model integrating a socioecological framework with health literacy at functional, interactive, and critical levels of enablement (38). Both make use of the visualization of health determinants as a “rainbow” of proximal and distal influences (39).

A further model identified in the review refers to an “equity-focused settings approach,” or “settings praxis” (40) that attends to health determinants, addresses the needs of marginalized groups, catalyzes change in a setting's structure, and involves stakeholders. The model engages with complexity theory to view settings as complex, decentralized systems that are organic, non-linear and emergent. It takes the form of a conceptual framework with six guiding principles: a holistic (whole-system) orientation; “start where people are”; place-based and joined-up practices; in-depth sociopolitical analysis; an asset-based approach; and a capabilities approach to health. Collectively, these principles position settings-as-systems in which health literacy, in systems terminology, is an active throughput.

Work on healthy universities (32) suggests that investigations into settings should identify the extent to which the setting promotes health through its policies and expressed purpose (core business). However, a successful settings-based approach, viewed from a systems perspective, is one of homeostasis: an ideal state of healthful, dynamic equilibrium whereby health becomes “business as usual” so seamlessly that it is difficult to evidence and separate out the settings-based approach as a factor (41). Using the settings-based approach to guide identification of the antecedents that need to be in place to constitute a supportive and enabling environment for health and health literacy therefore requires overcoming the challenge of documenting a successful settings-based approach within the system (i.e., when health literacy becomes systemic) (41). It is not within the remit of this review to resolve the challenge of evidencing a successful settings-based approach, only to conceptualize, based on evidence, what such an approach might entail.

### 3.3. Health-literate organizations

Another concept in the literature is health-literate organizations (HLOs), which by design support people to “systematically orient their daily routines toward HL [health literacy]” (42) (p. 464). HLOs “equitably enable individuals to find, understand, and use information and services to inform health-related decisions and actions for themselves and others” (43) (p. 1,084).

The HLO concept has several related terms, including organizational health literacy, health literacy-friendly organizations (6, 44, 45), and “organizational health literacy responsiveness” (15). Organizational health literacy “comprises a settings-based approach aiming at changing organizational conditions to enhance health literacy of relevant stakeholders” (46) (p. 1). It is underpinned by a set of auditable attributes of a “health-literate” organization (44, 47). There is potential for health-promoting settings and health-literate organizations to:

Work side by side (if not together), complementing each other […] Settings that have adopted a health promotion approach can easily become health literate settings and vice versa, because structures and processes have already been reoriented and important changes (including awareness) have already been implemented (48) (p. 888–889).

Organizational health literacy responsiveness is defined as:

The extent to which health workers, services, systems, organizations and policy-makers (across government sectors and through cross-sectoral public policies) recognize and accommodate diverse traditions and health literacy strengths, needs and preferences to create enabling environments that optimize equitable access to and engagement with health information and services, and support for the health and wellbeing of individuals, families, groups and communities (15) (p. x).

To date, the HLO concept has been mainly used with health care environments (47), and recently schools (46). To approach the public library as a HLO, or facilitate the process by which it can become one, requires a reorientation in the literature toward non-traditional or emerging settings without (yet) an accepted “health-promoting” or “health-literate” prefix, nor their own set of HLO principles. Part of such a reorientation would need to consider the antecedents for a setting to be considered an active, enabling, and responsive HLO.

### 3.4. The example case of the public library as part of a supersetting approach

The settings-based approach is “explicitly determinants-focused” (13) (p. 46). When implemented in line with this commitment, the approach changes the way people's environments are organized (13) and involves people in this change. It shares the Ottawa Charter's set of tenets that health promotion practice be enabling, participatory, holistic, intersectoral, equitable, sustainable, and use multiple strategies for health in combination (49). Based on these tenets, and the framework for settings praxis identified in the conceptual review (40), a determinants- and equity-focused settings approach is proposed and presented in a conceptual model that aims to engage with the complexity of health promotion interventions.

Conceptual models provide a reference-point for theorizing settings-based approaches and a reminder to attend to the interconnection between macro-, meso-, and micro-levels of a setting that inform a socioecological, whole-system perspective (32). The conceptual model here posits four antecedents that would need to be in place in, for example, a public library as a supportive and enabling environment that optimizes individuals' equitable access to and engagement with relevant health information and services (5, 18).

The identified antecedents are as follows:

A public library…

1. Acknowledges the wider determinants of health
2. Is open access
3. Involves local communities in how it is run
4. Facilitates informed action.

**Figure 1:**
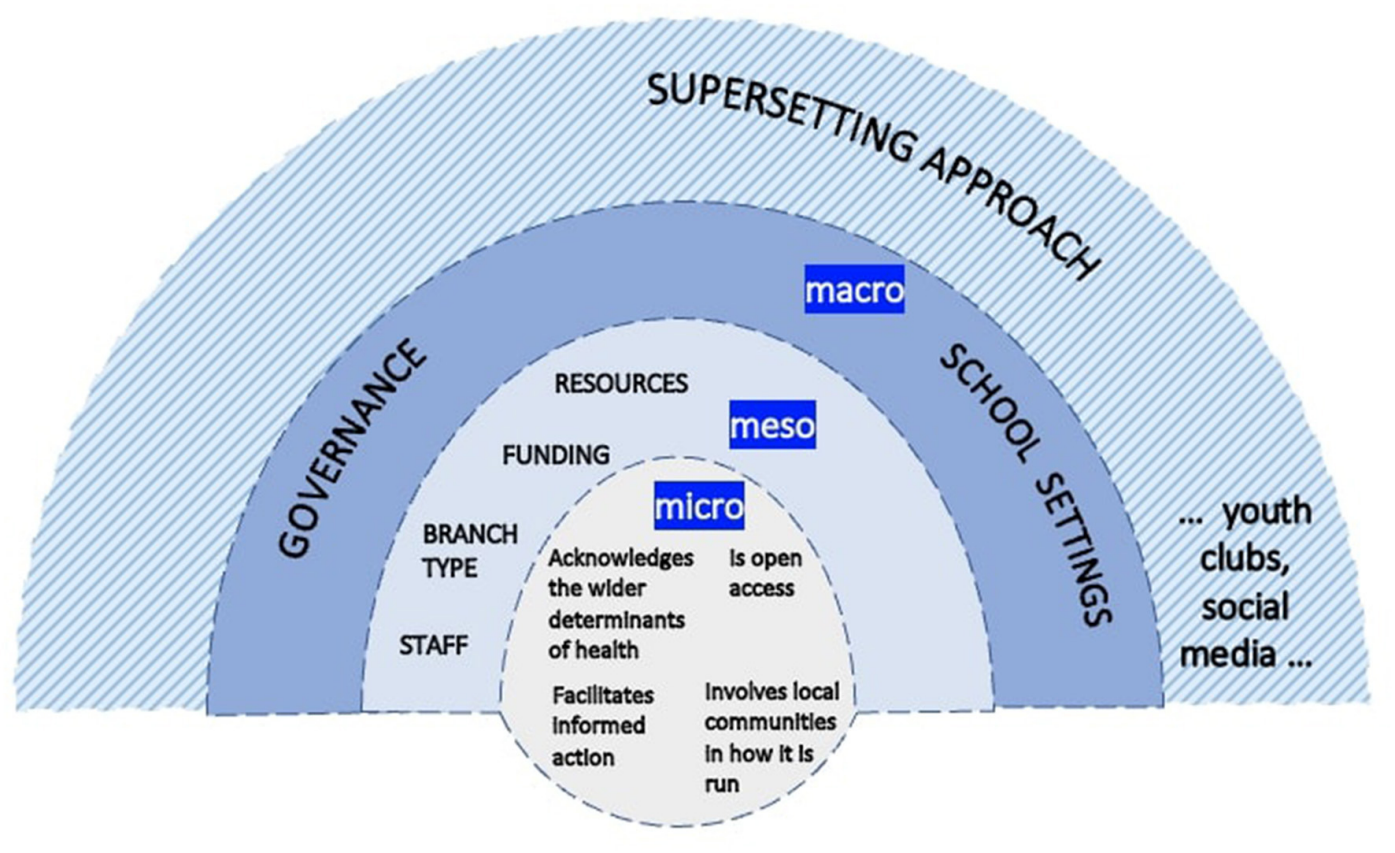
Conceptual model of the public library as part of a supersetting approach for health literacy development.

Figure 1 shows the conceptual model.

The model makes use of the visual shorthand of a “rainbow,” common to other models from the conceptual review (39), to represent the socioecological structure and operational levels of the public library. The library is shown as open to the wider environment (inputs from this environment include staff, funding, library branch facilities and resources; governance, policy, and school settings that influence library priorities; and additional potential partner settings for health). This is relevant to the tension between health promotion *in* a setting and a comprehensive settings approach: the partner settings depicted here are intended to support a comprehensive approach. Further example settings could include sports clubs and healthy universities, based on their steady emergence in the settings literature as non-traditional settings for health and health literacy (7, 9, 37, 50–53).

All four antecedents are holistic (i.e., they span the system) and work intersectorally (with porous boundaries to facilitate partnership work with other settings). In the example of the public library, all four antecedents are in place and position the library as part of a wider network of settings (or systems, or HLOs). The model demonstrates the “connectedness” (17) (p. 10) of this setting vertically (macro–micro) and horizontally (intersectoral collaboration across settings). The public library is strengthened through the participation of individuals interacting with it and other everyday settings over the life course (54): a supersetting approach.

The supersetting approach is a socioecological approach that builds on local knowledge and resources, is context-sensitive, and emphasizes participation (55). It is intended “to mobilize local communities for public health action through coordinated and integrated engagement of multiple stakeholders in multiple community settings” (56) (p. 2). It welcomes complexity (57) and recognizes the need to combine bottom-up, micro-level actions for health with (managed) top-down, macro-level influences (55) (p. 61).

The supersetting approach, as an intervention strategy for comprehensive community interventions, works through coordinated engagement of multiple stakeholders in multiple settings to mobilize local resources and support collective community action (55). It has five core principles: integration (coordinated action across specific settings); participation (people are motivated to take ownership of processes of developing and implementing activities); empowerment (there are opportunities for equity-focused action on authentic, relevant issues); context-sensitivity (people's everyday life challenges are respected and considered when developing and implementing activities); and knowledge generation and sharing (knowledge produced from coordinated activities is used to inform future activities) (17, 55, 57).

Advances in the supersetting approach are linked to Scandinavian public health research. The demonstration project SoL (from the Danish *Sundhed og Lokalsamfund*, “Health and Local Community”) marks the entry of the supersetting approach into the literature (17) and is the focus of several related papers. Citation analysis demonstrates that the public library is part of the historical development of the supersetting approach: *biblioteket* (library) appears as a label in a figure of the supersetting approach based on the 2014 original (58). By 2021, the presence of the library (joined also by “museum” and “sports club”) in the illustrative figure of the supersetting approach has passed into the English-language supersetting literature (59), separately from the Danish project SoL.

The supersetting approach can be linked with the settings concept of “projectism” (31) (p. 200), i.e., when “the theoretical framework guiding the work may be rooted in systems thinking and organizational development,” but practice is “constrained to smaller-scale project-focused work around particular issues” (60) (p. 56). Projectism is not incompatible with a comprehensive settings approach if the project—e.g., library-based health initiatives organized with partners—model “an inclusive and participative ethic” and “dynamic orientation” (21) (p. 893) commensurate with the supersetting approach.

## 4. Discussion

The conceptual review highlights how so-called non-traditional settings might support and enable the “project” of health literacy if this project is a collective endeavor, undertaken with support from other settings. It contributes a conceptual model of the public library (an example of a non-traditional setting) operating as part of a supersetting approach. The model theorizes the antecedents that need to be in place for the public library to be an enabling environment for health and health literacy in partnership with other settings, and points toward further areas for investigation.

To progress from health promotion *in* settings to active “settings for health,” the conceptual review proposes a coordinated supersetting approach. The supersetting approach is increasingly discussed in the literature (61, 62), but the most up-to-date handbook available for settings-based health promotion has few sustained discussions of it; the most substantive discussion refers to schools:

Actions in a school will be more effective when school activities are embedded in the local community, which will provide synergistic effects. This has been elaborated in the “supersetting” approach […] that summarizes sustainable approaches to optimized health, wellbeing and quality of life, and involves mobilizing the local community (63) (p. 109).

Many of the settings listed by the World Health Organization as settings for health do not have all the modeled antecedents consistently in place, including Health Promoting Schools (HPS). HPS are frequently represented in the literature as promoting health (30) and health literacy (64) early in the life course, but are limited in the support they can provide for facilitating “practical action” on health (4): children are not routinely encouraged to actively shape the school environment much beyond e.g., school council activities (65, 66). The conceptual model therefore includes schools, based on previous research into the settings that significantly influence library-based health promotion (18), but supplements this traditional setting with other settings that have different strengths and weaknesses in relation to the antecedents and penetrate people's lives at different stages of the life course.

Considering that health literacy is a setting-specific social practice (5), focusing on health literacy as a complex throughput in settings, and integrating settings into a supersetting approach that spans the life course (54), may advance population health literacy development and ensure that the settings concept continues to be relevant and responsive to future determinants of health.

## 5. Conclusion

This review has synthesized research and gray literature on settings from the 1980s to date. The results delineate how, despite the longstanding importance of settings for health and the settings approach in the development of health promotion and World Health Organization strategy, theorizing about settings remains under-developed (67). The review highlights some key conceptual challenges, including overlapping terms in the settings literature and theories from distinct disciplinary traditions (e.g., a systems perspective and health literacy responsiveness). The model developed from the conceptual review is helpful in providing a starter overview of antecedents to look for and in suggesting partnership opportunities between settings that collectively achieve the full complement of antecedents.

Understandings of the antecedents required so that settings can develop into settings for health and health literacy are advanced by a systems perspective and a supersetting approach that brings together multiple (traditional and non-traditional) settings to create and sustain supportive environments for health. This review has used the example of the public library to show the potential for a non-traditional setting for health, when part of a supersetting approach, to promote health and develop health literacy as “a whole-of-society endeavor—at the individual, community, and national level” that works “across sectors, not just health” (68). The direction of travel in a recent editorial in response to a World Health Organization report on health literacy development for the prevention and control of non-communicable diseases (5, 15), calling for “an integrative approach to develop health literacy interventions that involve a range of community-based organizations—not just medical centers—including schools, churches, sports groups, and workplaces” (68), is encouraging. But, in neither citing nor naming the supersetting approach that could potentially integrate such traditional and non-traditional settings, both the report and the editorial demonstrate the need to continue to review and refine the concept of settings.

## Author contributions

Conceptualization and writing—review and editing: CJ, SS, and JW. Writing—original draft preparation: CJ. Supervision: SS and JW. All authors have read and agreed to the published version of the manuscript.

## References

[1] Kickbusch I Gleicher D. Governance for Health in the 21st Century. Copenhagen: World Health Organization, Regional Office for Europe (2013). 107 p.

[2] World Health Organization. The Ottawa Charter for Health Promotion. WHO (1986). Available online at: http://www.who.int/healthpromotion/conferences/previous/ottawa/en/ (accessed March 21, 2020).

[3] Dooris M Kokko S Baybutt M. Theoretical grounds and practical principles of the settings-based approach. In:Kokko S Baybutt M, editors. Handbook of Settings-Based Health Promotion. Cham: Springer International Publishing (2022). p. 23–44 10.1007/978-3-030-95856-5_2

[4] World Health Organization. Health Promotion Glossary of Terms 2021. Geneva: World Health Organization (2021). Available online at: https://apps.who.int/iris/handle/10665/350161 (accessed December 11, 2021).

[5] World Health Organization. Health Literacy Development for the Prevention and Control of Noncommunicable Diseases: Volume 1: Overview. Geneva: World Health Organization (2022). https://apps.who.int/iris/handle/10665/364203 (accessed November 15, 2022).

[6] Okan O Kirchhoff S Bauer U. Health literate schools (HeLit-Schools): organizational health literacy in the school setting. Eur J Public Health. (2021) 31:56. 10.1093/eurpub/ckab164.14533001212

[7] Baybutt M Kokko S Crimeen A de Leeuw E Tomalin E Sadgrove J. Emerging settings. In:Kokko S Baybutt M, editors. Handbook of Settings-Based Health Promotion. Cham: Springer International Publishing (2022). p. 225–38.

[8] Jenkins CL. A student perspective on learning and doing settings-based health promotion in the era of TikTok. In:Akerman M Germani ACCG, editors. International Handbook of Teaching and Learning in Health Promotion: Practices and Reflections From Around the World. Cham: Springer (2022). p. 733–44. 10.1007/978-3-030-96005-6_45

[9] Levin-Zamir D Bertschi IC McElhinney E Rowlands G. Digital environment and social media as settings for health promotion. In:Kokko S Baybutt M, editors. Handbook of Settings-Based Health Promotion. Cham: Springer International Publishing (2022). p. 205–24.

[10] Tolentino M Millerd S Bali NZ Ranido E Takiguchi J Balaz HJ. Next Gen Hawai‘i: collaborative COVID-19 social media initiative to engage Native Hawaiian, other Pacific Islander, and Filipino Youth. Hawaii J Health Soc Welf. (2022) 81:201–8. 35821668 PMC9272528

[11] TikTok Cultures Research. Health #foryou?: health education communities on TikTok. TikTok Cultures Research (2020). Available online at: https://tiktokcultures.com/2020/11/02/health-foryou/ (accessed May 16, 2021).

[12] Nutbeam D. What would the Ottawa Charter look like if it were written today? Crit Public Health. (2008) 18:435–41. 10.1080/09581590802551208

[13] Dooris M. Expert voices for change: bridging the silos—towards healthy and sustainable settings for the 21st century. Health Place. (2013) 20:39–50. 10.1016/j.healthplace.2012.11.009 23376729

[14] Kickbusch I. NCDs in today's Europe. in Fifth Meeting of the Regional Director's Advisory Council on Innovation for NCDs. Berlin: EUPHA (2022).

[15] World Health Organization. Health Literacy Development for the Prevention and Control of Noncommunicable Diseases: Volume 2. A Globally Relevant Perspective. Geneva: World Health Organization (2022). Available online at: https://www.who.int/publications/i/item/9789240055353 (accessed November 15, 2022).

[16] Newman L Baum F Javanparast S O'Rourke K Carlon L. Addressing social determinants of health inequities through settings: a rapid review. Health Promot Int. (2015) 30(Suppl. 2):ii126–43. 10.1093/heapro/dav054 26420808

[17] Bloch P Toft U Reinbach HC Clausen LT Mikkelsen BE Poulsen K. Revitalizing the setting approach – supersettings for sustainable impact in community health promotion. Int J Behav Nutr Phys Act. (2014) 11:118. 10.1186/s12966-014-0118-8 25218420 PMC4172849

[18] Jenkins CL Sykes S Wills J. Public libraries as supportive environments for children's development of critical health literacy. Int J Environ Res Public Health. (2022) 19:11896. 10.3390/ijerph191911896 36231198 PMC9564910

[19] Paakkari O Paakkari L. Health literacy as a learning outcome in schools. Health Educ. (2012) 112:133–52. 10.1108/09654281211203411

[20] Lenstra N McGehee M. Public librarians and public health: how do partners perceive them? J Library Outreach Engag. (2022) 2:66–80. 10.21900/j.jloe.v2i1.883

[21] Whitelaw S Coburn J Lacey M McKee MJ Hill C. Libraries as ‘everyday' settings: the Glasgow MCISS project. Health Promot Int. (2017) 32:891–900. 10.1093/heapro/daw021 27006366

[22] Philbin MM Parker CM Flaherty MG Hirsch JS. Public libraries: a community-level resource to advance population health. J Community Health. (2019) 44:192–9. 10.1007/s10900-018-0547-4 29995303 PMC6329675

[23] Leung R, Flaherty, MG, Rudd, R, Toumbourou, JW,. Are Libraries Effective Settings for Accessing Health Information?: An Evidence Check Rapid Review. The Sax Institute for the NSW Ministry of Health (2016). Available online at; https://www.saxinstitute.org.au/evidence-check/are-libraries-effective-settings-for-accessing-health-information/ (accessed December 18, 2022).

[24] St. Jean B, Jindal G, Liao Y, Jaeger PT, eds. Roles and Responsibilities of Libraries in Increasing Consumer Health Literacy and Reducing Health Disparities. Bingley: Emerald Group Publishing (2021). 10.1108/S0065-2830202047

[25] Kyabaggu R Marshall D Ebuwei P Ikenyei U. Health literacy, equity, and communication in the COVID-19 era of misinformation: emergence of health information professionals in infodemic management. JMIR Infodemiol. (2022) 2:e35014. 10.2196/35014 35529308 PMC9066383

[26] Luo L Park VT. Preparing public librarians for consumer health information service: a nationwide study. Library Inform Sci Res. (2013) 35:310–7. 10.1016/j.lisr.2013.06.002

[27] Whiteman ED Dupuis R Morgan AU D'Alonzo B Epstein C Klusaritz H. Public libraries as partners for health. Prev Chronic Dis. (2018) 15:1–9. 10.5888/pcd15.170392 29806580 PMC5985906

[28] ALLIANCE. Co-Creating Libraries for Wellbeing Report. Health and Social Care Alliance Scotland (2021). Available online at: https://www.alliance-scotland.org.uk/blog/resources/co-creating-libraries-for-wellbeing-report/ (accessed October 21, 2021).

[29] Naccarella L Horwood J. Public libraries as health literate multi-purpose workspaces for improving health literacy. Health Promot J Australia. (2020) 32:29–32. 10.1002/hpja.437 33140444

[30] World Health Organization. Healthy Settings. WHO. Available online at: https://www.who.int/teams/health-promotion/enhanced-wellbeing/healthy-settings (accessed August 13, 2020).

[31] Whitelaw S Baxendale A Bryce C MacHardy L Young I Witney E. ‘Settings' based health promotion: a review. Health Promot Int. (2001) 16:339–53. 10.1093/heapro/16.4.339 11733453

[32] Dooris M Wills J Newton J. Theorizing healthy settings: a critical discussion with reference to Healthy Universities. Scand J Public Health. (2014) 42:7–16. 10.1177/1403494814544495 25416568

[33] Dooris M Kokko S de Leeuw E. Evolution of the settings-based approach. In:Kokko S Baybutt M, editors. Handbook of Settings-Based Health Promotion. Cham: Springer International Publishing (2022). p. 3–22 10.1007/978-3-030-95856-5_1

[34] Ayala RA. Thinking of conceptual reviews and systematic reviews. Nurs Inquiry. (2018) 25:e12264. 10.1111/nin.12264 30325098

[35] Hulland J. Conceptual review papers: revisiting existing research to develop and refine theory. AMS Rev. (2020) 10:27–35. 10.1007/s13162-020-00168-7

[36] Wenzel E. A comment on settings in health promotion and public health. Internet J Health Promot. (1997). Available online at: https://ldb.org/setting.htm

[37] Kokko S. Sports clubs as settings for health promotion: fundamentals and an overview to research. Scand J Public Health. (2014) 42:60–5. 10.1177/1403494814545105 25416575

[38] Dawkins-Moultin L McDonald A McKyer L. Integrating the principles of socioecology and critical pedagogy for health promotion health literacy interventions. J Health Commun. (2016) 21:30–5. 10.1080/10810730.2016.1196273 27668970

[39] Dahlgren G Whitehead M. The Dahlgren-Whitehead model of health determinants: 30 years on and still chasing rainbows. Public Health. (2021) 199:20–4. 10.1016/j.puhe.2021.08.009 34534885

[40] Shareck M Frohlich KL Poland B. Reducing social inequities in health through settings-related interventions – a conceptual framework. Glob Health Promot. (2013) 20:39–52. 10.1177/1757975913486686 23797939

[41] Dooris M. Healthy settings: challenges to generating evidence of effectiveness. Health Promot Int. (2006) 21:55–65. 10.1093/heapro/dai030 16339774

[42] Nowak P Dietscher C Sator M. Health literacy policies: national example from Austria – a unique story and some lessons learned from an ongoing journey. In:Okan O Bauer U Levin-Zamir D Pinheiro P Sørensen K, editors. International Handbook of Health Literacy: Research, Practice and Policy Across the Lifespan. Bristol: Policy Press (2019). p. 453–70. 10.51952/9781447344520.ch030

[43] Brach C Harris LM. Healthy People 2030 health literacy definition tells organizations: make information and services easy to find, understand, and use. J Gen Intern Med. (2021) 36:1084–5. 10.1007/s11606-020-06384-y 33483812 PMC8042077

[44] Koh HK Baur C Brach C Harris LM Rowden JN. Toward a systems approach to health literacy research. J Health Commun. (2013) 18:1–5. 10.1080/10810730.2013.759029 23305507 PMC5127593

[45] Meggetto E Ward B Isaccs A. What's in a name? An overview of organisational health literacy terminology. Aust Health Rev. (2017) 42:21–30. 10.1071/AH17077 29117893

[46] Kirchhoff S Dadaczynski K Pelikan JM Zelinka-Roitner I Dietscher C Bittlingmayer UH. Organizational health literacy in schools: concept development for health-literate schools. Int J Environ Res Public Health. (2022) 19:8795. 10.3390/ijerph19148795 35886647 PMC9316432

[47] Brach C Keller D Hernandez LM Baur C Parker R Dreyer B. Ten Attributes of health literate health care organizations. NAM Perspect. (2012) 1–26. 10.31478/201206a

[48] Gugglberger L. The multifaceted relationship between health promotion and health literacy. Health Promot Int. (2019) 34:887–91. 10.1093/heapro/daz093 31755534

[49] Evaluation in Health Promotion : Principles and Perspectives. Denmark: World Health Organization (2001).

[50] Johnson S Van Hoye A Donaldson A Lemonnier F Rostan F Vuillemin A. Building health-promoting sports clubs: a participative concept mapping approach. Public Health. (2020) 188:8–17. 10.1016/j.puhe.2020.08.029 33049492

[51] Geidne S Kokko S Lane A Ooms L Vuillemin A Seghers J. Health promotion interventions in sports clubs: can we talk about a setting-based approach? A systematic mapping review. Health Educ Behav. (2019) 46:592–601. 10.1177/1090198119831749 30795690

[52] Newton J Dooris M Wills J. Healthy universities: an example of a whole-system health-promoting setting. Glob Health Promot. (2016) 23:57–65. 10.1177/1757975915601037 27199018

[53] Paakkari L Kokko S Villberg J Paakkari O Tynjälä J. Health literacy and participation in sports club activities among adolescents. Scand J Public Health. (2017) 45:854–60. 10.1177/1403494817714189 28673131

[54] Whitehead D. Before the cradle and beyond the grave: a lifespan/settings-based framework for health promotion. J Clin Nurs. (2011) 20:2183–94. 10.1111/j.1365-2702.2010.03674.x 21535458

[55] Magnus E Knudtsen MS Wist G Weiss D Lillefjell M. The search conference as a method in planning community health promotion actions. J Public Health Res. (2016) 5:jphr.2016.621. 10.4081/jphr.2016.621 27747199 PMC5062752

[56] Jourdan D Christensen JH Darlington E Bonde AH Bloch P Jensen BB. The involvement of young people in school- and community-based noncommunicable disease prevention interventions: a scoping review of designs and outcomes. BMC Public Health. (2016) 16:1123. 10.1186/s12889-016-3779-1 27784301 PMC5080716

[57] Grabowski D Aagaard-Hansen J Willaing I Jensen BB. Principled promotion of health: implementing five guiding health promotion principles for research-based prevention and management of diabetes. Societies. (2017) 7:10. 10.3390/soc7020010

[58] von Heimburg D, Hofstad, H,. Samskaping som samarbeids- og styringsform for kommunenes folkehelsearbeid: Hva vet vi? Hva er utfordrende? Og hvor går vi videre? Oslo: Oslo Metropolitan University (2019). Available online at: https://oda.oslomet.no/oda-xmlui/bitstream/handle/20.500.12199/1328/2019-11.pdf?sequence=1 (accessed November 24, 2022).

[59] Tørslev MK Andersen PT Nielsen AV Petri M Termansen T Vardinghus-Nielsen H. Tingbjerg Changing Diabetes: a protocol for a long-term Supersetting initiative to promote health and prevent type 2 diabetes among people living in an ethnically and socioeconomically diverse neighbourhood in Copenhagen, Denmark. BMJ Open. (2021) 11:e048846. 10.1136/bmjopen-2021-048846 34580094 PMC8477325

[60] Dooris M. Joining up settings for health: a valuable investment for strategic partnerships? Critical Public Health. (2004) 14:49–61. 10.1080/09581590310001647506

[61] Mikkelsen BE Bloch P Reinbach HC Buch-Andersen T Lawaetz Winkler L. Project SoL—A community-based, multi-component health promotion intervention to improve healthy eating and physical activity practices among danish families with young children part 2: evaluation. Int J Environ Res Public Health. (2018) 15:1513. 10.3390/ijerph15071513 30021938 PMC6069463

[62] Toft U Bloch P Reinbach HC Winkler LL Buch-Andersen T Aagaard-Hansen J. Project SoL—a community-based, multi-component health promotion intervention to improve eating habits and physical activity among Danish families with young children. Part 1: intervention development and implementation. Int J Environ Res Public Health. (2018) 15:1097. 10.3390/ijerph15061097 29843434 PMC6025396

[63] St.Leger L, Buijs G, Keshavarz Mohammadi N, Lee A. Health-promoting schools. In:Kokko S Baybutt M, editors. Handbook of Settings-Based Health Promotion. Cham: Springer International Publishing (2022). p. 105–17 10.1007/978-3-030-95856-5_6

[64] World Health Organization. Shanghai Declaration on Promoting Health in the 2030 Agenda for Sustainable Development. WHO (2016). 10.1093/heapro/daw103 28180270

[65] Ioannou S Kouta C Charalambous N. Moving from health education to health promotion: developing the health education curriculum in Cyprus. Health Educ. (2012) 112:153–69. 10.1108/09654281211203420

[66] Jensen BB. Environmental and health education viewed from an action-oriented perspective: a case from Denmark. J Curric Stud. (2004) 36:405–25. 10.1080/0022027032000167235

[67] Wills J Sykes S Hardy S Kelly M Moorley C Ocho O. Gender and health literacy: men's health beliefs and behaviour in Trinidad. Health Promot Int. (2019) 26(Suppl. 1):i29–69. 10.1093/heapro/daz076 31407795

[68] The Lancet. Why is health literacy failing so many? Lancet. (2022) 400:1655. 10.1016/S0140-6736(22)02301-7 36366878

